# Computed Tomography Study of the Mummy of King Seqenenre Taa II: New Insights Into His Violent Death

**DOI:** 10.3389/fmed.2021.637527

**Published:** 2021-02-17

**Authors:** Sahar N. Saleem, Zahi Hawass

**Affiliations:** ^1^The Department of Radiology, Kasr Al Ainy Faculty of Medicine, Cairo University, Cairo, Egypt; ^2^Former Minister of Antiquities of Egypt, Cairo, Egypt

**Keywords:** mummy, Egypt, mummification, Hyksos, computed tomography (CT), royal mummies, Seqenenre Taa II

## Abstract

Seqenenre-Taa-II, The Brave, (c.1558–1553 BC) ruled Southern Egypt during the occupation of Egypt by the Hyksos. The mummy was physically examined and X-rayed in the 1960s, which showed severe head wounds that have prompted various theories about the circumstances of his death. We postulated that Computed Tomography (CT) study of Seqenenre-Taa-II's mummy would give insights into the circumstances of his death. We examined Seqenenre's mummy using CT and compared the findings with the archaeological literature as well as with five Asian weapons found in Tell-el-Dabaa. CT findings indicate that Seqenenre died in his forties. The mummies deformed hands suggest that the King was likely imprisoned with his hands tied. CT images provided detailed analysis of Seqenenre's previously reported injuries to the forehead, right supra-orbital, nose-right orbit, left chick, and skull base. This study revealed additional craniofacial fractures in the right lateral side of the skull that had been concealed by the embalmers beneath layers of material. Analysis of the morphology of the injuries enabled a better understanding of the mechanism of trauma, possible number of the attackers, and their relative position to the King. The size and shape of the fractures correlated well with the studied Hyksos weapons. The lethal attack was aimed at the King's face, likely in an attempt to disgrace him. Mummification of Seqenenre's body was limited to evisceration without brain removal. The desiccated brain is shifted to the left side of the skull. This may indicate that the King's dead body stayed on its left side for some time—long enough for decomposition start before the mummification began. This suggests that the King likely died at a location distant from the funeral place, possibly on a battlefield. The embalmers attempted to conceal the King's injuries; the methods used suggest that the mummification took place in a royal mummification workshop rather than in a poorly equipped location. CT findings of Seqenenre's mummy helped us to better understand the circumstances of his violent death. His death motivated his successors to continue the fight to unify Egypt and start The New Kingdom.

## Introduction

Seqenenre-Taa-II was the Egyptian king who ruled Southern Egypt at the end of the 17th Dynasty (approximately1558–1553 BC), during the Hyksos occupation of Egypt. In the ancient Egyptian language, “Heqau-Khasut” meant “the rulers of the foreign lands”; the word was transmitted by Greek sources as “Hyksos.” The Hyskos were likely a group of Asian shepherds who occupied the Northern part Egypt and took Avaris (modern Tell el Dabaa) as the capital for a period of time called the “second intermediate period” (c. 1650–1550 BC). Although the Egyptian rulers maintained power over the South (capital Thebes), all of Egypt had to pay tributes to the Hyksos King (1, 2).

An ancient Papyrus, The Sallier I; Papyrus I, stated that there was hostility between Seqenenre and the king of Hyksos, Apophis. According to the Papyrus, the Hyksos king Apophis sent a hostile message to Seqenenre, stating that noisy hippopotamuses in a pool in Thebes were disturbing his sleep in Avaris (644 Km away), and demanding that the Theban sacred pool be destroyed. The end of the Papyrus is lost, but the preserved text ends with the statement that Seqenenre called his counselors, which probably indicates an introduction to a battle (2).

Deir el-Ballas, a settlement just north of Thebes, was likely the base for military campaigns against the Hyksos. Text from an ostraca which dates to the 17th dynasty, found at Deir el-Ballas, indicates that a large number of men, large quantities of goods, and ships with their crews were brought to the site. A lintel of Seqenenre, also found at Deir el-Ballas, confirmed that the site was founded during his reign. A stela known as The Carnavaron Tablet, found in Thebes Karnak Temple, recorded the battles that Kamose, Seqenenre's son, fought against the Hyksos in the North (3–5). Kamose fell dead during the war against the Hyksos and it was Ahmose, the second son of Seqenenre, who completed the expulsion of the Hyksos, chased them to Sharuhen (modern Ghaza in Palastine), and unified Egypt (6). However, there has been no record of the fate of Seqenenre.

In 1881 at the Deir el-Bahari cache in Thebes, a mummy was discovered in its original linen wrappings and was transferred to Cairo Museum. On June 9th, 1886, the mummy was unwrapped by Gaston Maspero (the general director of antiquities in Egypt) accompanied by Daniel Fouquet (a physician). Maspero identified the mummy as Seqenenre-Taa by the inscriptions on the original wrappings. Taa (or Tao) was the birth name and meant “Thoth is great”; while “Seqenenra” was the throne name and meant “The one whom Ra has made brave” (7). Maspero and Fouquet reported a foul odor of the putrefied mummy, limited body mummification, as well as severe head injuries suggestive of a violent death (7, 8). On September 1st, 1906, Grafton Elliot Smith, the professor of anatomy at Kasr Al Ainy School of Medicine in Cairo, examined the mummy of Seqenenre. Smith described the mummy's head injuries in detail, and noted the absence of wounds on the arms or on the rest of the body (9). In the 1960s, the X-ray study of Seqenenre's mummy confirmed five separate traumatic injuries confined to the head, and the absence of any fractures to the rest of the skeleton (10).

Several scenarios were proposed to explain how Seqenenre's head injuries, though questions remained regarding the circumstances of Seqenenre's death. Had the King died in battle? Was he a victim of a palace conspiracy? (6, 11). Was Seqenenre's body hastily mummified in a poorly equipped location away from the royal mummification workshop? (6).

Computed Tomography (CT) is a non-invasive modality that has been used to examine the mummies of several ancient Egyptian royals and has allowed for greater insight into the mysterious circumstances under which they died (6).

We postulated that a CT study of mummy Seqenenre, in correlation with the historical data, could give more insights on his death circumstances and could shed new light on this important chapter in Egypt's history.

## Materials and Methods

**Figure 1:**
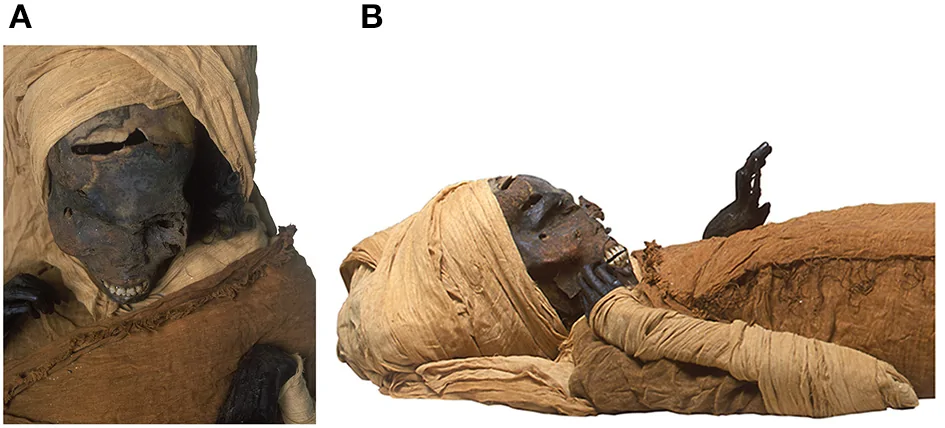
Picture of mummy Seqenenre-Taa-II. **(A)** Picture of head and upper torso of the mummy Seqenenre-Taa-II shows severe multiple craniofacial injuries. **(B)** Picture of right lateral view of the head and upper torso of mummified Seqenenre shows the deformed upper limbs with flexed hands at the wrists and spastic fingers.

The mummy of Seqenenre is located at Cairo Egyptian Museum with the catalog code [JE 26209(b) CG 61051 SR1/10192] (Figure 1).

On the 4th of May 2019 we transferred the mummy of Seqenenre to the multi-detector Computed Tomography (CT) scanning machine (Somatom Emotion 6; Siemens Medical Solutions, Malvern, Pennsylvania) installed on a truck in the garden of the Cairo Egyptian Museum.

We used the following CT parameters: kVp = 130 effective mAs ranged from 23 to 63; pitch ranged from 0.83 to 1.8; FOV from 350 to 500; slice thickness from 0.6 to 1.25 mm; and reconstruction from 0.4 to 0.8 mm. Axial images were created. We used a separate workstation (Leonardo Workstation, Siemens Medical Solution) to reconstruct the CT images in multiple two-dimensional planes, as well as a three-dimensional reconstruction. We analyzed the CT images of the mummy to assess the preservation status, age at death, and pathologies. We also examined five Asian bronze weapons housed in the Cairo Egyptian Museum, originally found in Tell el Dabaa region by the Austrian Archaeological Mission. The weapons originated during the Hyksos period (Middle Bronze II), which was concurrent with Seqenenre's reign (12). Each weapon was photographed and measured. We retrieved the available data on each weapon from the Cairo Egyptian Museum's files as well as from previous research studies (12). We correlated CT findings of the mummy of Seqenenre with the weapons as well as with the available archaeological data.

## Results

**Figure 2:**
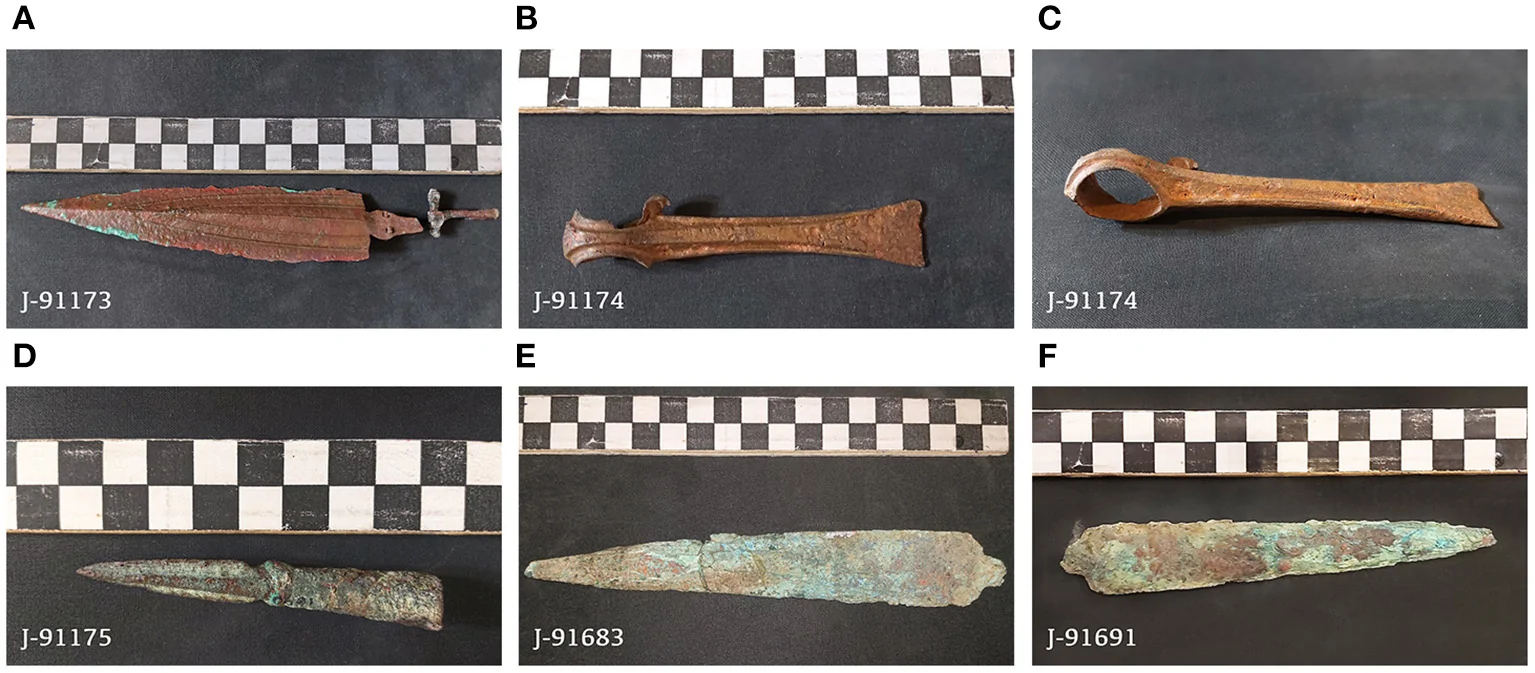
Photographs of bronze Asian weapons MB II (Middle Bronze II era) found in Tell el Dabaa (ancient Avaris). N.B. The accompanying codes are those given by Cairo Egyptian Museum where the weapons are currently housed. Each square of the measuring ruler equals to 10 mm in length. **(A)** Dagger (J-91173): A corroded fractured dagger with five-lined blade. Three nails are seen on the broken handle-thorn. **(B,C)** Battle-ax (J-91174) front and side views of a battle-ax. The cutting edge of the blade is straight. The hollow handle is in line with the cutting blade; this gives the weapon more stability and force. **(D)** Spearhead (J-91175) A triangular blade with a well-defined mid rib that extends along the full length of the blade to a thick shank. The blade has a rhomboid cross section. A large blade, shank, and tang form (tripartite). Tang to get into the shaft, and a shank to be used as stop and partial support for binding. **(E)** Bronze dagger (Code: J-91683) from MB II (Middle Bronze II era) found in Tell el Dabaa-Egypt (ancient Avaris). **(F)** Bronze dagger (Code: J-91691) from MB II (Middle Bronze II era) found inTell el Dabaa (ancient Avaris).

Table 1 gives the results of our examination of five Asian bronze weapons housed in the Cairo Egyptian Museum that were originally found in Tell el Dabaa region. We inspected three daggers, a battle-ax, and a spearhead (Figure 2). Previous studies indicated that these weapons belong to the Asian Middle Bronze Age Culture II, which coincided with Seqenenre's reign (12).

**Table 1:** Weapons found in Tell E-Dabaa (Ancient Avaris) belonged to Hyksos (Middle Bronze Culture II-MB II) at the time of Seqenenre's reign.

**Registered code**	**Type**	**Description**	**Measurements**
J-91173	Dagger	Bronze dagger corroded and fractured. Dagger blade has five lines and diamond-shaped in cross section. Three nails on the handle-thorn are broken	Full Length of object: 230 mm; Breadth: 38 mm; Length of blade 18.3 mm
J-91174	Battle ax	Narrow bladed shaft-hole bronze battle-ax in corroded condition. The handle is in line with the blade. The hollow handle is rounded at the upper end and oval at the lower side. The middle of the ax is having hexagonal cross section. The cutting edge is straight.	Length of the object: 142 mm, Breadth of the cutting edge: 27.5 mm
J-91175	Spear head	A triangular blade with well-defined mid rib that extends the length of the blade to a thick shank. The blade is diamond in shape in cross section.	Full Length of the object: 104 mm, Length of the blade 60 mm, maximum Breadth 15 mm
J-91683	Dagger	Long dagger. Lenticular in cross section	Length 215 mm, breadth 35 mm
J-91691	Dagger	Long dagger. Lenticular in cross section	Length 282 mm, breadth 30 mm

### CT Study of the Mummy

#### Preservation Status

The mummy of Seqenenre is in poor condition; the head and many bones are loose and misplaced. The mummy is disarticulated in most body regions. The head is separate from the body. Most of the vertebrae and ribs are loose. The sternum, clavicles and scapulae are disarticulated and located inside the torso cavity. The upper limbs are disarticulated from the shoulders with minimal soft tissues or muscles remaining on the bones. Both femora are disarticulated from the hip sockets. The bones of the left forefoot are disarticulated. The symphysis pubis and the right sacro-iliac joints are disrupted.

#### Age at Death

We estimated the age at death to be around 40 years based on epiphyseal closure of all long bones, fusion of the meta-and meso-sterna, and symphysis pubis surface features (stage 6 corresponding to 35–39 years) (13). There is a complete set of teeth inside the mouth, including all of the third molars. Mild to moderate attrition of the teeth is observed: grade G in the mandibular teeth (corresponding to 35–40 years), and grade H in the maxillary teeth (corresponding to 40–50 years) (14, 15).

#### Stature

We could not measure the length of the skeleton in its articulated anatomical position because of its partly disarticulated status. However, we measured the maximum length of the tibia (384 mm) to calculate stature using the regression equation derived by Raxter et al. for ancient male Egyptians: (Stature = 2.554 × 38.4 + 69.12 = 167.2 cm ± 3.002). We thus estimated the stature of Seqenenre to be about 167 cm ± 3 cm (16).

#### Mummification

**Figure 3:**
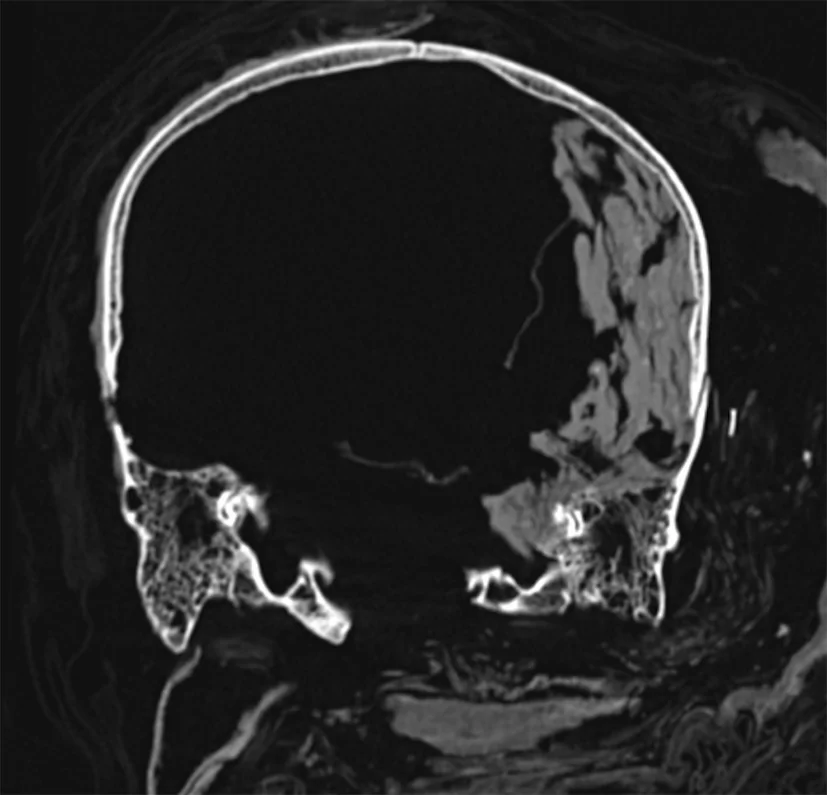
Coronal CT image of the head and neck of mummy “Seqenenre-Taa-II.” The desiccated brain is seen shifted and occupying the left side of the cranial cavity.

**Figure 4:**
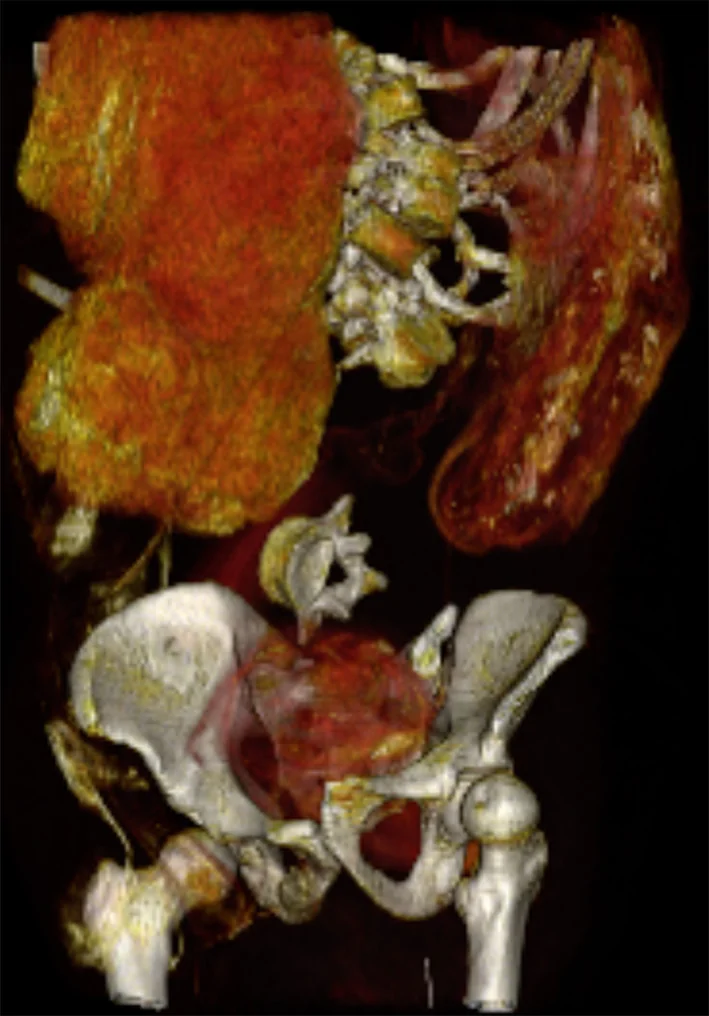
Coronal 3-dimensional CT image of lower torso of “Seqenenre-Taa-II” shows evisceration and embalming material within the abdomino-pelvic cavity. Note the disarticulated vertebrae, and disrupted symphysial articulation.

The desiccated shrunken brain occupies the left side of the cranial cavity. There is no intracranial resin or other evidence of other embalming materials. The cribriform plate is intact with no CT evidence of attempt of brain removal (Figure 3). There is no evidence of heart or viscera inside the body cavity. The abdominal and pelvic cavities are stuffed with packs of variable CT densities consistent with linen (−100 HU) and resin (70–120 HU) (Figure 4). A left flank opening measuring 157 mm in length was likely used to remove the viscera and insert the packs. No amulets or jewelry could be detected within the mummy or on its surface.

#### Pathological Changes

**Figure 5:**
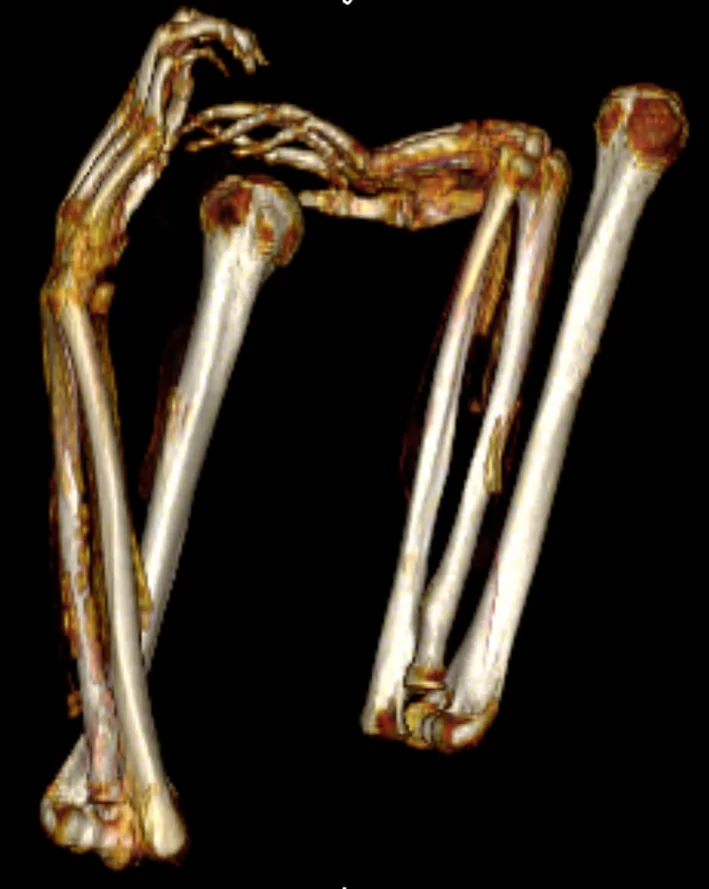
Three-dimensional frontal CT image of upper limbs of mummy of Seqenenre-Taa-II. The arms are dislocated at the shoulder and bent at the elbows. Note the deformity of the bent hands bent indicating they were tied at the wrists.

Both arms are flexed at the elbows, the hands are flexed at the wrists (more on the left side), while the fingers are hyper-extended at the metacarpo-phalangeal joints and flexed at the inter-phalangeal joints. No evidence of bony fractures in upper limbs was seen (Figure 5). Both lower limbs are extended; the left foot is dislocated but there is no evidence of bony fractures. The head and face are severely injured. The upper and lower lips are retracted, showing the teeth.

### CT of Head Injuries, Causative Weapons, and Position of the Assailants

**Figure 6:**
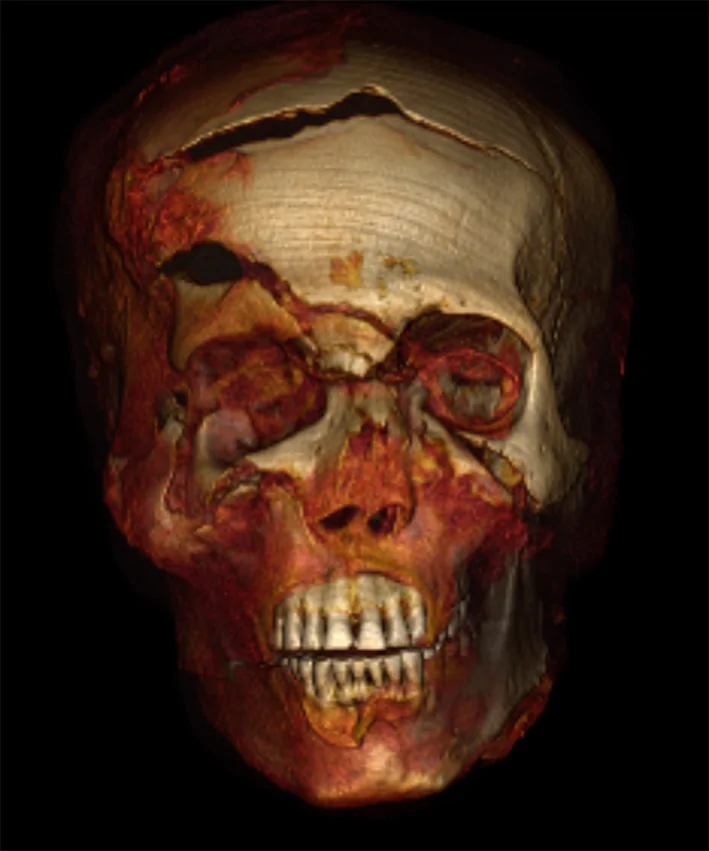
Three-Dimensional CT image of head of Seqenenre in frontal projection showing multiple craniofacial injuries: a gaping fracture of the frontal bone; fracture above the right eyebrowe; blunt trauma caused comminuted fractures of the nose, right orbit, and right zygoma; and a small perforating wound overlying the right cheek caused by fragments of the fractured zygoma.

**Figure 7:**
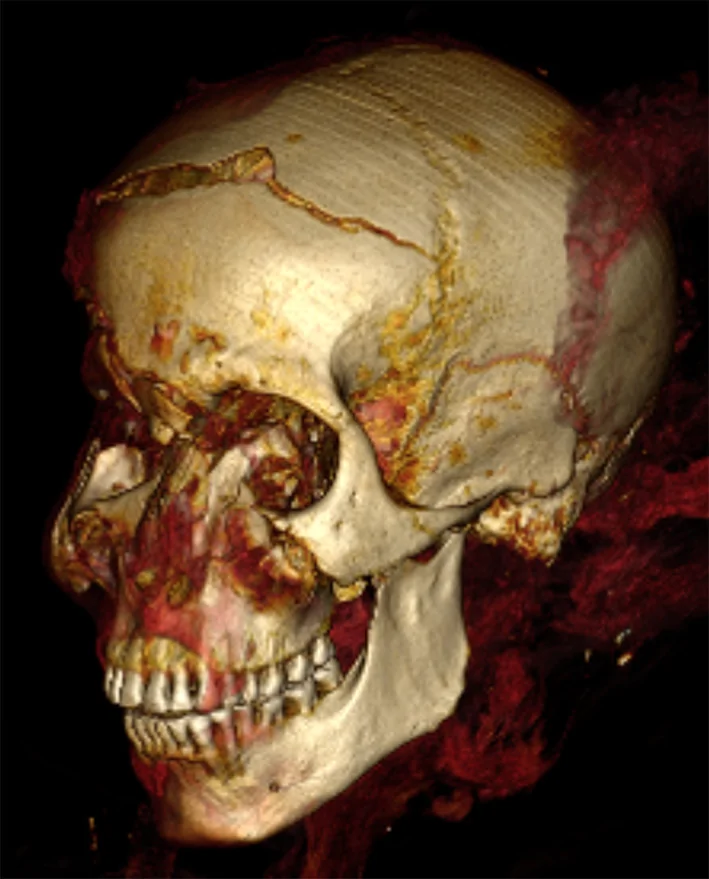
Three-Dimensional CT image of Seqenenre's head in left oblique projection shows an oblique cut wound of the left zygoma, fracture of left coronoid process of the mandible.

**Figure 8:**
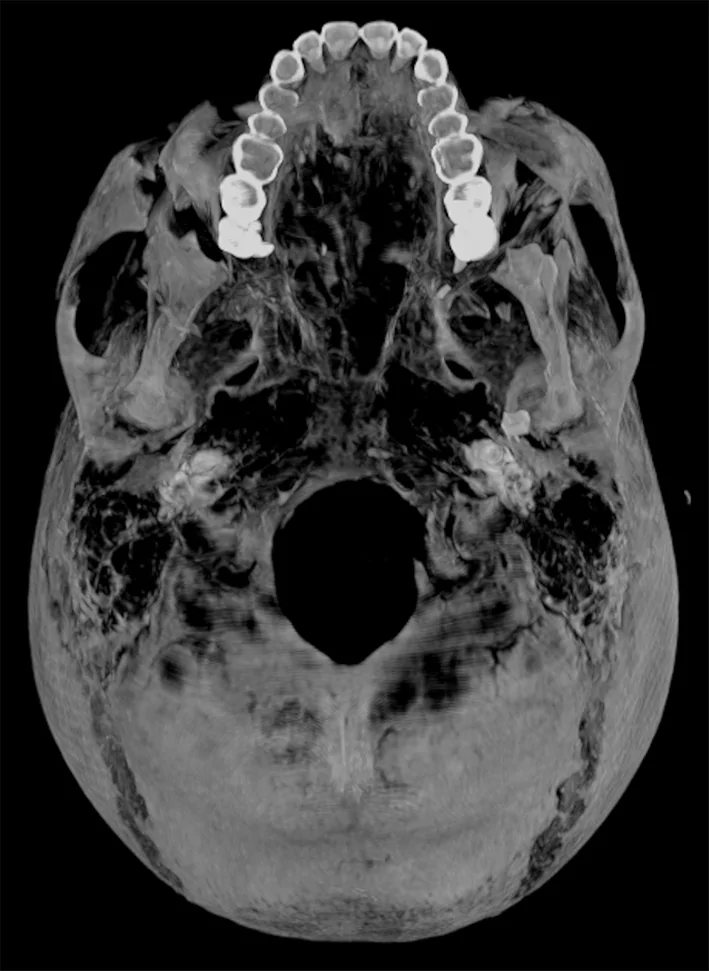
Axial thick slab three-dimensional reconstructed computed tomography image of the external view of the skull base of Seqenenre's mummy showing fracture of the left lip of foramen magnum inflicted by a penetrating injury of the left mastoid bone.

Figures 6–8 and Supplementary Figures 1, 2 show the head injuries.

#### A Cut Fracture in the Upper Forehead Region

##### Description

The upper region of the frontal bone shows a transverse cut fracture that starts 5 mm to the left of the midline and extends transversely to the right for 70 mm. The gapping fracture is of irregular width that varies between 4 and 14 mm, being wider on the left side. A secondary fissure fracture extends horizontally for 43 mm from the left end of the original fracture line.

##### Suggested Weapon

A heavy sharp object like a sword or an ax. The blow was most likely fatal.

##### Position of the Assailant

The site of the fracture at the top of Seqenenre's head indicates that the assailant was positioned above the King. This relative positionality may have been achieved by the assailant being in a raised position, e.g., riding a horse, or by the King being in a lowered position, either sitting down or kneeling. The assailant was most probably in front of the King on his left side. This assumption is based on the width of the cut fracture and its site; the cut fracture being wider at the left side indicates that the stronger impact came from the left side.

#### Right Supra-Orbital Puncture Wound

##### Description

A spindle-shaped gaping fracture is seen just above the right supra-orbital margin. It extends almost horizontally for 32 mm. The fracture has narrow ends and a wider central part that measures 12 mm. This gapping fracture is associated with a short fissure fracture that extends from its medial end obliquely toward the fractured glabella.

##### Suggested Weapon

A double-edged blade weapon, its dimensions correspond to the wound's measurements (about 32 mm wide and 12 mm thick). The injury may fit with the bronze battle-ax (MB II) found in Tel el-Dabaa (Figures 2B,C).

##### Position of the Assailant

The blow came perpendicularly from an assailant standing in front of Seqenenre toward his right side. The blow was most likely fatal.

#### Injuries of the Nose, the Right Orbit, and the Right Cheek Bone

##### Description

###### Fractured glabella.

An almost horizontal fissure fracture of the glabella measures 23 mm in length and 3 mm in width. The fracture involves the anterior and posterior tables of the frontal sinus. The right edge of the glabella fracture joins the medial end of the previously described right supraorbital fracture.

###### Fractured nose.

A transverse fracture of the nasal bridge 5 mm caudal to the naso**-**frontal suture. The fracture lines gap for 6 mm. Both nasal bones are displaced posteriorly for 8 mm, deviating to the right, and show multiple comminuted fractures. Also the cartilaginous elements and soft tissues of the nose are distorted and shifted to the right side.

###### Fractured zygoma.

A gapping vertical fracture 22 mm in length splits and displaces the body of the right zygoma posteriorly. It is possible that the displaced fractured zygoma prevented translation of the coronoid process and resulted in a locked jaw. A zygomatico-maxillary complex fracture is also seen, composed of fractures of the zygomatic arch, inferior orbital rim, anterior and posterior maxillary sinus walls, as well as the lateral orbital rim. The sharp fragments of the comminuted fracture of the right fronto-zygomatic process penetrated the overlying skin and resulted in an oval hole (measures 12 mm craniocaudal and 7 mm transversely).

##### Suggested Weapon

The described injuries (in the nose and the right side of the face) were likely caused by blunt forcible blow (or multiple blows) using a heavy blunt object (e.g., a thick stick/the handle of ax).

##### Position of the Assailant

The blow came from the front of the King toward his right side, diagonally and from above.

#### Left Cheek Region

##### Description

An oblique cut fracture of the left side of the face extends for 35 mm from the left orbit lower margin in the direction of the zygomatic-maxillary suture. The cut fracture involves the body of the left zygoma as well as the tip of the coronoid process of the left mandible. The corresponding cut wound in the skin is spindle in shape with two pointed ends and a gapping center for 5 and 17 mm in depth.

##### Suggested Weapon

A heavy sharp weapon such as a sword or an ax. The bronze battle-ax found in Tel-el Dabaa (Figure 2B) fit into the size and the spindle shape of the described wound.

##### Position of the Assailant

The blow came from the front of the King and to his left side.

#### Injury to the Left Side of the Skull Base

##### Description

Irregular transverse fracture of the lower part of the mastoid (with the tip of the mastoid missing), fractured left occipital condyle, and the left side of the margin of the foramen magnum. The fracture extends for 35 mm.

##### Suggested Weapon

A pointed long sharp weapon similar to the Hyksos spearhead found at Tel el Dabaa (Figure 2D)**. **The sharp spearhead penetrated the left mastoid transversely toward the foramen magnum. This wound could be fatal as it may have caused injury of the brainstem-spinal cord.

##### Position of the Assailant

The blow came horizontally from the left side of Seqenenre below his left ear.

### Injuries of the Right Lateral Side of the Skull

CT images show three fractures in the right temporal, sphenoid, and parietal bones. These fractures are mostly covered by embalming material and cannot be detected by physical inspection.

#### Right Temporal Fracture

##### Description

A transverse cut fracture of the squamous part of the right temporal bone runs parallel and about 5 mm inferior to the parieto-temporal suture. It joins anteriorly with the fractured sphenoid and extends posteriorly for about 55 mm. The fracture is 5–6 mm wide with a short central constriction that measures about 2 mm. A short vertical fracture 23 mm in length extends caudally from the anterior edge of the fracture.

##### Suggested Weapon

A sharp weapon with a cut section matching the fracture's shape and size such as a dagger in Figures 2E,F.

##### Position of the Assailant

The blow came horizontally from the right side of the King.

#### Fractured Greater Wing of Right Sphenoid

##### Description

The CT images reveal comminuted fracture of the greater wing of the right sphenoid bone. The fracture shows as a roughly triangular region lateral and posterior to the right orbit. The narrow apex points downwards, with the top of the triangle being the base, measuring 17 mm. The cranio-caudal height is about 20 mm.

##### Suggested Weapon

A heavy sharp object like a dagger like in Figure 2E or Figure 2F.

##### Position of the Assailant

The blow came horizontally from the right side of the King.

#### A Right Parietal Fracture

##### Description

A vertical fissure fracture runs from above downward in the anterior part of the right parietal bone; it measures 30 mm in length and 3 mm in width. At the lower end, the fracture turns posteriorly for a short segment 13 mm in length and 2 mm in width (possibly a secondary fracture).

##### Suggested Weapon

The blow came vertically from the right side of the King. Possibly the king was lying down on his left side.

##### Position of the Assailant

Most probably the weapon used to inflict the injury was a heavy blunt object like a thick stick.

There is no CT evidence of healing in any of the described craniofacial fractures. Few small broken bone chips are seen, having fallen within the cranial cavity.

## Discussion

The CT study of the mummified Seqenenre shows that he was in his forties when he died. This age estimate is in accordance with the historical resources (10). The physical examination of Seqenenre's mummy done by GE Smith in 1906 (9), as well as the 1960s X-rays, both also estimated that the King died at age 40 (10). However, as neither physical examination nor plain x-ray allow for examination of the mummy's pubic symphyseal face, age estimation was based on less reliable criteria such as degenerative bony changes and closure of the cranial sutures (9, 10). In this study, we were able to make a more accurate estimation of the mummy's age at death using the three-dimensional CT morphology of the pubic symphyseal face. The undulating surface of the pubic symphyseal face becomes smoother with age, allowing for improved accuracy of age estimation (15).

Because of the disarticulated skeleton of the mummy, it is not possible to directly measure vertex to heel stature. Early in the twentieth century, GE Smith reassembled Seqenenre's mummy and estimated the stature to be about 170 cm (9). In this CT study, we measured the tibial length and extrapolated the stature of Seqenenre using a regression equation derived for data for ancient male Egyptians to be 167 ± 3cm (16). King Seqenenre Taa's stature is comparable to the height of most of the New Kingdom Kings: Thutmose III's stature was 165 cm, Tutankhamun 167 cm, Seti I 167 cm, Ramesses II 172 cm, Merenptah 172 cm, and Ramesses III 165 cm (17).

The most striking feature in the mummy of Seqenenre is the presence of severe injuries in the head (1, 9, 10). Multiple craniofacial injuries in Seqenenre's mummy were previously described in the literature based on physical inspection and X-rays (9, 10). In 1898, Maspero described three injuries by physical inspection involving the upper forehead, the right cheek and the left cheek (7). GE Smith in 1912 described two other injuries: one near the nose region and one on the left side of the skull base (9).

CT imaging using two and three-dimensional reconstruction facilitates in-depth visualization of the bony and soft tissue elements of a mummy (17). In this study, CT imaging allowed for detailed visualization of Seqenenre's previously reported head injuries, beyond what was possible using only x-rays or under physical inspection. The injuries involved five different regions: a transverse fracture of the forehead, a gaping fracture above the right eyebrow, a blunt trauma to the nose and right eye region, a sharp cut wound of the left cheek, and a penetrating wound below the left ear through the skull base.

CT also revealed additional injuries to the right side of the skull that were not described in the previous studies (7, 9, 10). These injuries were partially hidden beneath layers of embalming material. We suggest that the embalmers deliberately concealed these injuries, likely as a desperate attempt to beautify the injured corpse of the King.

A blunt trauma or a sharp force trauma may result in skeletal fractures. Analysis of the morphology of the fracture is often informative to the type of trauma, and the direction of the impact. The shape and size of the cut fracture reflects the blade of the weapon that induced it: the wider the cut fracture, the larger the blade. Variation in the width of the cut fracture indicates the direction of the impact: the wider part of the cut fracture points to the side of the stronger impact (18). The morphology and location of Seqenenre's injuries suggest that several aggressors attacked him using different weapons. However, the scholars have not been in agreement regarding the type of weapons that caused Seqenenre's injuries. According to Maspero, the left cheek injury was caused by a heavy stick (a club), the right cheek injury was inflicted by a dagger, and the forehead by an ax (7). In contrast, GE Smith believed that an ax or a sword caused the forehead, right supra-orbital, and left cheek injuries, a heavy stick (or the handle of an ax) inflicted the nose injury, and a spear was used to penetrate the skull base (9).

Scholars did agree that the morphology of Seqenenre injuries matched those that would be inflicted with weaponry from the Hyksos period (2, 12, 19). Bietak and Strouhal studied Asian bronze weapons found in Tell El Dabaa (ancient Avaris) and matched them to Seqenenre's head injuries. The weapons belonged to the Middle Bronze Culture II (MB II), and were similar to those used by Hyksos authorities at the time of Seqenenre's reign (12). We studied five of these Asian bronze weapons, currently housed at Cairo Egyptian Museum. We agree with Bietak and Strouhal's suggestion that the left cheek and right supra-orbital fractures match with the size and shape of MB II battle-ax (Figures 2B,C). The cutting edge of the Hyksos battle-ax is smaller than its Egyptian counterpart. The force of the impact of the Hyksos ax is more focused and impacts a narrower area, explaining the deep bone punctures we observed (2, 12, 20).

We believe the forehead fracture was caused by a weapon with a broader blade than the battle-ax which caused the left cheek and the right supra-orbital injuries. According to Bietak and Strouhal, a broad-edged ax caused the forehead facture, which could have been an Egyptian weapon (12).

This study suggests that a thick stick (the handle of a battle ax) caused the blunt fracture around the King's nose. We believe that the left mastoid and skull base injury may have been caused by the studied MB II spearhead. We confirm in this CT study that Seqenenre's craniofacial injuries were inflicted around the time of the King's death (perimortem) as there is no evidence of healing. Such severe craniofacial trauma could have caused fatal shock, blood loss, and/or intracranial trauma.

The variety in attack angle, as well the wide range of weapons we believe to have caused the King's injuries, indicate that Seqenenre was killed in battle by numerous enemy attackers. The match between weapons and the morphology of the injuries strongly suggests that Seqenenre was killed during a war between the Egyptians and the Hyksos (2). The location of Seqenenre's craniofacial injuries indicates that he was likely facing his attackers. Facial injuries in combat are usually paired with fractures of the forearms (parry fractures), as it is a natural reflex to throw up the arms to protect the face (21). However, this CT study confirms that Seqenenre's forearms were not fractured and that there are no injuries elsewhere in the body. This conclusion has been also suggested by previous X-ray studies done in the 1960s (10).

Seqenenre's mummified hands are flexed at the wrists and the fingers are spastic. This arm position is not consistent with the usual crossed-pectoral position of the royal ancient Egyptian mummies. This hand position suggests that the King's wrists were tied together, likely behind the body, when he died. If the King's died while his hands were bound, the muscles that were in intense contraction just before death would have become rigid immediately after death, unable to relax; this condition is known as cadaveric spasm. It typically affects the hands and limbs of individuals who were subjected to violent deaths and whose nervous systems were disturbed at the moment death, e.g., a drowning victim's hand clenching on weeds from the waterbed. A deceased body demonstrating a cadaveric spasm will maintain that position until the body decomposes (22). Cadaveric spasm is not part of rigor mortis, the progressive stiffness of the deceased muscles that is seen over the course of a few hours after death due to natural biomechanical changes (22–24). Cadaveric spasm has been considered as a possible explanation in other archeological contexts: in an ancient Roman fortification (300–400 AD), a male with a head trauma was found holding a pig molar between his clenched fingers (23). A scenario in which Seqenenre's hands were tied during his attack and subsequent death would also explain the absence of defensive injuries on the King's arms, as he would have been unable to shield or protect himself from his assailants (6).

Scholars have proposed several possible scenarios for the King's death. In 1912, GE Smith suggested that the King was killed while lying down on his right side (9). Others have added that this attacked likely took place while he slept in his palace (8, 10). They hypothesize that because all of the craniofacial injuries were inflicted horizontally from aggressors at the King's left side, he must have been sleeping on his right side at the time of the attack (9, 12). In 1974, Bietak and Strohal used the results of their physical examination and 1960's skull x-ray to argue that the King was riding a chariot into battle at the moment of his death. They assumed that Seqenenre received the first blow to his left cheek from an attacker below him. When Seqenenre lowered his head, possibly to look at his attacker, he received the second blow that landed just above his right eye. The King's head sagged down as a result, which allowed the third blow to hit his forehead and the fourth blow to smash the base of the nose (12). A final explanation was posited by Shaw in 2009, who argues that Seqenenre was imprisoned and executed by the victorious Hyksos (6).

We believe that a situation in which the King was attacked while sleeping is unlikely, as the locations of the injuries demonstrate that the blows came not only from the left side of the King but also from other directions. A blow from the right side resulted in the right supra-orbital fracture, one from above caused the forehead fracture, and strikes from the front left fractured the left cheek and the left mastoid (12). Furthermore, if the King died in the palace, then the body would have been preserved properly. However, when Maspero unwrapped Seqenenre's mummy, he reported greasy bandages and bad odor, leading to his suggestion that the body must have been already decomposing by the time of mummification (1). Scholars have therefore reasoned that the corpse of Taa decomposed during the journey to Thebes from the location of his death and was mummified as soon as it arrived (6). This theory is supported by the location of the King's brain, shown in this CT study. The desiccated brain is usually seen occupying the posterior region of the cranial cavity in mummies lying down in the supine position (24). However, in this case, as decomposition of the dead body progressed, the brain shifted toward the most dependent region. CT images in this study show Seqenenre's desiccated brain shifted toward the left side of the cranial cavity. This is consistent with the results of a recent CT study of a mummy with a head tilted to the right side, which showed that the shrunken brain was also shifted to the right of the skull cavity (25). We therefore suggest that the King's dead body must have remained in a left lateral position for some time; perhaps on the battlefield where he died, or while the mummy was being transported to Thebes. It is unlikely that Seqenenre's body was placed in the lateral position during the process of mummification, as embalmers at that time usually placed the body in a neutral supine position (2).

Though we agree with Bietak and Strohal (12) that the King likely died as a result of a battle with the Hyksos, we believe that Shaw's theory (2) best explains the results of this CT study. We argue that Seqenenre fought the Hyksos, was captured, and that his hands were cuffed (6). We agree with other scholars that there is no evidence for the definite order of the blows that King Seqenenre received, or which one killed him (2, 6, 9, 12). Seqenenre received several lethal wounds: the forehead, the right supra-orbital, and possibly the skull base. Any one of those injuries is a potential cause of death, but the three of them together is almost certainly fatal. We assume that the King was at a lower position than his assailant(s), possibly kneeling at least for some time during the attack. This position explains the high forehead injury that could have been the first blow the king received, inflicted by a sword or an ax. The strong hit must have caused the King to fall down, possibly on his back. The King may have received several attacks from the assailant with the Hyksos battle ax, possibly using its blade to inflict the fracture above the right eyebrow (right supra-orbital). Then a thick stick (possibly the handle of the ax) was used to smash the nose and the right eye of the King. The assailant hit the King's left side of the face with the ax. Another assailant at the left side used a spear horizontally to pierce deeply the lower part of the left ear (mastoid) and reached the foramen magnum. We assume that the King was dead at this point, and that his body was rolled to lie at his left side where he received several blows to the right side of the skull possibly by a dagger. The dead King likely stayed lying down on his left side for some time enough for the body to start decomposition as the brain shifted to this dependent side.

The exact location of the King's death is unknown, as there is no definite historic evidence for the location of a battle between Seqenenre and the Hyksos (3). However, Deir el-Ballas, located just north of Thebes, was a base built by Seqenenre to launch military campaigns against the Hyksos (4, 5). We assume that a battlefield would have been located somewhere between the Egyptian stronghold at Deir el-Ballas and the Hyksos capital Avaris, and that Seqenenre's dead body was likely transferred from the battlefield to Thebes *via* Deir el-Ballas. The location at which Seqenenre's body was then mummified is a question of great scholarly focus. Some scholars have suggested that Seqenenre's body was mummified hastily on the battlefield (1, 9). Another plausible explanation would place his mummification at Deir el-Ballas, as excavations at the site have uncovered a palace and tombs which date to Seqenenre's time (26). However, there is no evidence that intentional mummification procedures took place at Deir el-Ballas: there were no canopic jars found there and all of the human remains were rather skeletons (26). This CT study demonstrates that the embalmers went to great lengths to properly mummify the body of Seqenenre. They attempted to conceal some of Seqenenre's head injuries using a paste of embalming materials, a purely cosmetic procedure which highlights the care with which the King was mummified. Though the embalmers did not attempt to remove the brain of Seqenenre, this seems to have been the norm for other royals, such as those who dated to the early 18th Dynasty like Thutmose II (1493–1479 BC) and Thutmose III (1479–1425 BC) (26). The embalmers would have been unable to place the mummy in the usual neutral supine position with the arms crossed at the chest due to the partial decomposition and the cadaveric spasm of his hands. We therefore do not agree with previous scholarship that Seqenenre's body was hastily or improperly mummified. We believe that Seqenenre's corpse was primarily mummified in the Theban royal mummification workshop, and not at in a poorly equipped or temporary setting as others have argued.

It seems that Seqenenre was well-prepared for the war against Hyksos as he founded the huge military fortress at Deir el-Ballas. He probably took the provocation of Hyksos King as a justification to declare war. When compared to CT scanning of other ancient Egyptian warriors with great military reputations, such as Thutmose III and Ramesses II, only Seqenenre had severe injuries inflicted by weapons (17). Such injuries suggest that Seqenenre was on the front line, risking his life to liberate Egypt. Seqenenre's death motivated his successors to continue the fight to unify Egypt and start the New Kingdom.

## Conclusion

This study of the mummified body of King Seqenenre-Taa-II helped to develop a better understanding of the circumstances of his death. This study suggests that King Seqenenre was likely killed while leading the Egyptian military against the Hyksos army. He was captured in battle and was killed in the struggle.

The reconstructed CT images enabled a detailed analysis of the King's craniofacial injuries, helped match the injuries with the weapons used to inflict them, and suggest a possible scenario of the attack.

## Data Availability Statement

The original contributions presented in the study are included in the article/Supplementary Material, further inquiries can be directed to the corresponding author/s.

## Ethics Statement

The studies involving human participants were reviewed and approved by the Egyptian Ministry of Antiquities and Tourism Committee. Written informed consent for participation was not required for this study in accordance with the national legislation and the institutional requirements.

## Author Contributions

SS was responsible for the conception and design, acquisition of data, analysis and interpretation of data, as well as drafting of the manuscript, generation of the figures, and was accountable for accuracy and integrity of the work. ZH made substantial contributions to the design, interpretation of the results, drafting of the manuscript, critical revisions for important intellectual content, and agreed to be accountable for the integrity of any part of the work. Both authors read and approved the manuscript.

## Conflict of Interest

The authors declare that the research was conducted in the absence of any commercial or financial relationships that could be construed as a potential conflict of interest.

## Abbreviations

AD: Anno Domini
BC: Before Christ
cm: centimeter
CT: Computed Tomography
DNA: deoxyribonucleic acid
FOV: Field-Of-View
HU: Hounsfield Unit
KV: Kilovolt
mAS: milliampere-seconds
MIP: Maximum Intensity Projection
mm: Millimeter
MPR: Multiplanar Reconstruction
SSD: Surface shaded display
VRT: volume rendering technique.
