# Supplementary material for: Computed Tomography Study of the Mummy of King Seqenenre Taa II: New Insights Into His Violent Death
Source: Front Med (Lausanne). 2021 Feb 17;8:637527. doi: 10.3389/fmed.2021.637527 (PMC7925410; doi:10.3389/fmed.2021.637527)

Supplementary Figure 1:

Photograph of the front face of mummified Seqenenre Taa II obtained in June 2020 shows the facial injuries


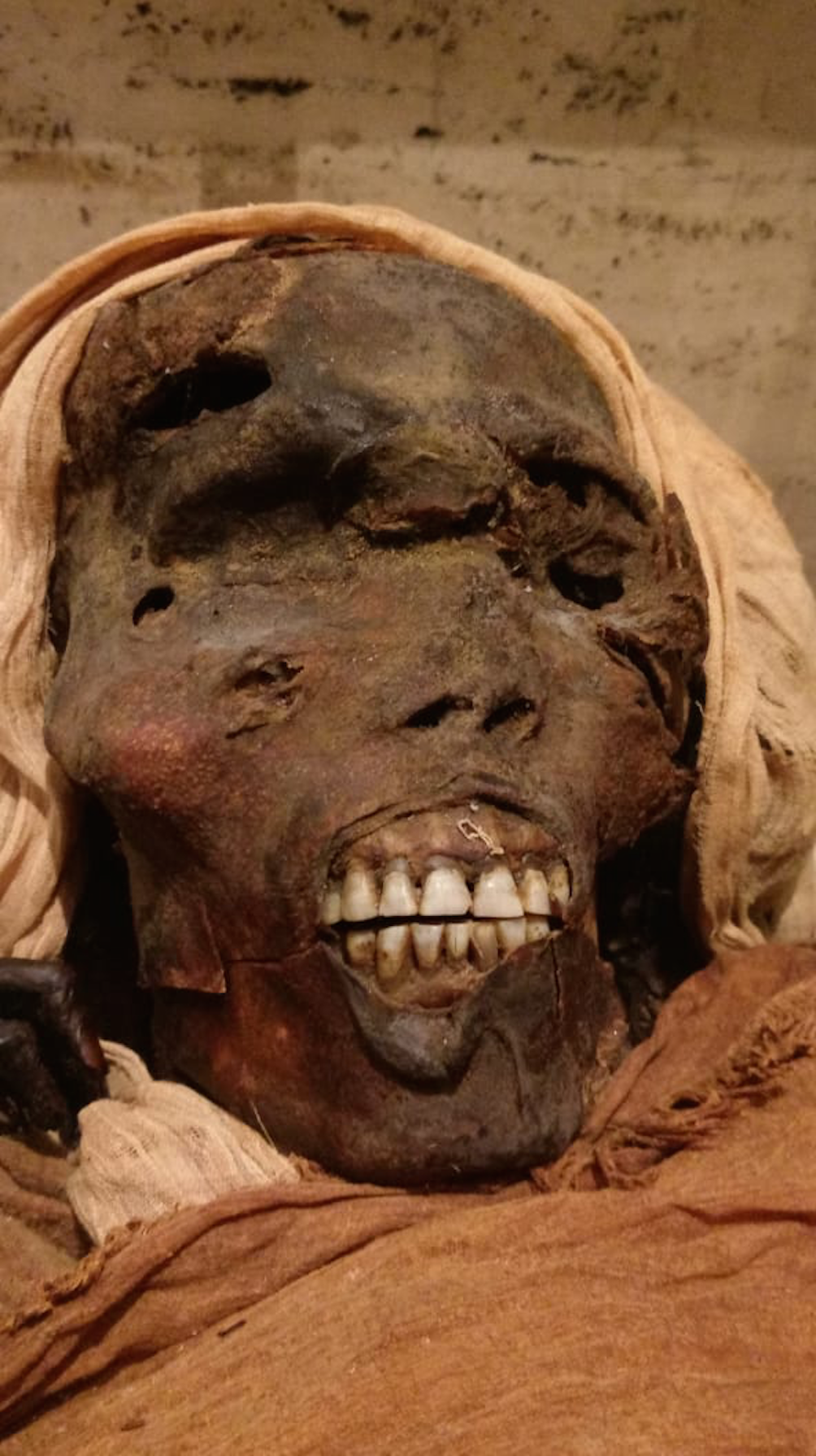


Supplementary Figure 2:

Left oblique photograph of the face of mummified Seqenenre Taa II obtained in June 2020 shows the facial injuries


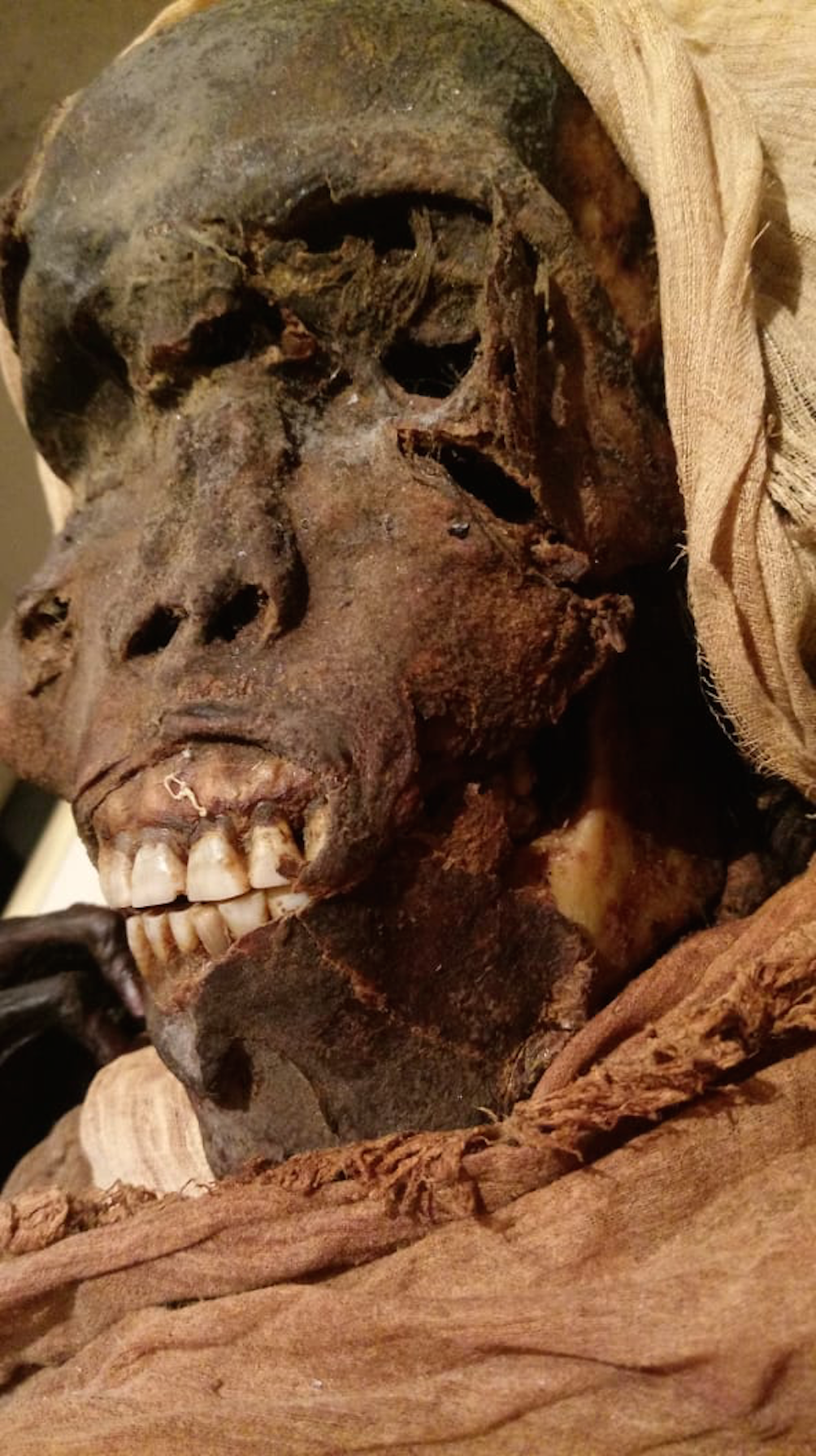

Supplement: Supplementary file 1 [file Table_1.DOCX]
